# Raeding with the fingres: Towards a universal model of letter position coding

**DOI:** 10.3758/s13423-022-02078-0

**Published:** 2022-06-01

**Authors:** Ana Baciero, Pablo Gomez, Jon Andoni Duñabeitia, Manuel Perea

**Affiliations:** 1 Faculty of Science and Technology, Department of Psychology, Bournemouth University, Fern Barrow, BH12 5BB Poole, UK; 2 California State San Bernardino, Palm Desert Campus, Palm Desert, CA USA; 3 Centro de Investigación Nebrija en Cognición, Universidad Antonio de Nebrija, Campus Madrid – Princesa, C/ Santa Cruz de Marcenado, 27, 28015 Madrid, Spain; 4 The Arctic University of Norway, Tromsø, Norway; 5 Universitat de València, Valencia, Spain

**Keywords:** Transposed-letter effect, Word recognition, Braille, Lexical decision

## Abstract

Letter position coding in word recognition has been widely investigated in the visual modality (e.g., labotarory is confusable with laboratory), but not as much in the tactile modality using braille, leading to an incomplete understanding of whether this process is modality-dependent. Unlike sighted readers, braille readers do not show a transposed-letter similarity effect with nonadjacent transpositions (e.g., labotarory = labodanory; Perea et al., 2012). While this latter finding was taken to suggest that the flexibility in letter position coding was due to visual factors (e.g., perceptual uncertainty in the location of visual objects (letters)), it is necessary to test whether transposed-letter effects occur with adjacent letters to reach firm conclusions. Indeed, in the auditory modality (i.e., another serial modality), a transposed-phoneme effect occurs for adjacent but not for nonadjacent transpositions. In a lexical decision task, we examined whether pseudowords created by transposing two adjacent letters of a word (e.g., laboartory) are more confusable with their base word (laboratory) than pseudowords created by replacing those letters (laboestory) in braille. Results showed that transposed-letter pseudowords produced more errors and slower responses than the orthographic controls. Thus, these findings suggest that the mechanism of serial order, while universal, can be shaped by the sensory modality at play.

## Introduction

When reading in alphabetic writing systems, orthographic processing (i.e., encoding the identity and order of the letters) acts as the interface between perceptual and linguistic processing (see Grainger, 2018, for review). As such, it is a topic of great interest for researchers in word recognition and reading, mainly in the visual modality. In this context, a considerable wealth of visual-word recognition experiments has shown that letter position coding is only approximate. In lexical decision experiments (*is the stimulus a word or not?*), pseudowords generated by transposing two letters of a word, whether adjacent or nonadjacent (e.g., JUGDE, CHOLOCATE) are more easily confusable with their base words (JUDGE, CHOCOLATE) than replacement-letter controls (e.g., JUPTE, CHOTONATE). This finding, referred to as the *transposed-letter [similarity] effect* (e.g., Perea & Lupker, 2004), has been consistently reported in a variety of languages (e.g., English, Spanish, French, Thai, Hebrew, etc.). Of note, a parallel effect occurs when transposing two characters in non-alphabetic writing systems (e.g., syllabaries such as Japanese, or logograms such as Chinese).

The flexibility of letter position coding in the visual modality, as attested by the transposed-letter effect, served to rule out those visual-word recognition models with a strict scheme for letter position coding (e.g., McClelland & Rumelhart's, 1981, interactive-activation model and its successors). Note that if letter position within a letter string were encoded with precision, one would expect similar performance for JUDGE and JUPTE (or CHOLOCATE and CHOTONATE). Furthermore, the robustness of the transposed-letter effect drove researchers to design more refined and flexible models of the front-end of visual-word recognition. There are two leading families of these models. First, the *perceptual* accounts (e.g., Davis, 2010; Gomez et al., 2008; Norris & Kinoshita, 2012) suggest that there is uncertainty (or noise) associated with the position of the letters within a word due to the inherent limitations of the visual system; therefore, each of the letters of a word would activate not only its own position but also other nearby positions. Second, the *orthographic* accounts (e.g., Grainger & van Heuven, 2003; Whitney, 2001) state that the order of letters within a word is coded later in processing, at a literacy-dependent level where linguistic information is stored as letter pairs. Specifically, these models assume a processing level between the letter and word levels composed by open bigrams (i.e., contiguous and non-contiguous ordered letter pairs). The more open bigrams two strings of letters share, the greater the perceptual similarity between them (see also Duñabeitia et al., 2015, for an orthographic account without relying on open bigrams). Of note, other models opt for combining these two approaches (e.g., Adelman, 2011; Grainger et al., 2006). Despite the many studies conducted to examine the predictions of these accounts (e.g., Davis & Lupker, 2017; Marcet et al., 2019; Massol et al., 2013), the debate is still very much alive today.

It has recently been postulated that the fundamental mechanisms behind the encoding of letter order in reading are shared with other serial order processes (e.g., serial recall or typing), operating under the different constraints posed by each task (see Fischer-Baum, 2018; Houghton, 2018; Logan, 2021). To test the universality of this claim, here we examined a fundamental marker of letter position coding (i.e., the transposed-letter effect) in the tactile modality during braille reading. Furthermore, the analysis of this issue will also help separate the general properties of the word recognition system from those that result from the limitations of the specific sensory system that collects information (see Fischer-Baum & Englebretson, 2016, for an instance of the same rationale applied to morphological processing).

In the following, we present an overview of the braille system. Then, we review the scarce literature on letter position coding in braille, and, finally, we introduce the rationale of the experiment. The braille writing system was created by Louis Braille around 200 years ago (based on Barbier’s alphabet; see Braille, 1829). It is a system of embossed dots whose basic unit is *the cell,* an array of two by three dots. The different configurations of elevated dots form the elements of the written language such as letters (e.g., a =  ) or punctuation marks (e.g., ? =  ). A total 2^6^ = 64 combinations of raised dots can be configured in a cell (International Council on English braille, 2013). Reading braille involves scanning the text from left to right with the fingertips. This motion creates a shear force that is sensed by the mechanoreceptors innervating the finger (see Gardner & Johnson, 2013). Such haptic stimulation yields a serial sensory experience – at least when using one finger – that contrasts to the more parallel nature of word recognition in the visual modality.^1^

To our knowledge, only two studies have examined letter position coding in braille. Perea et al. (2012) reported a lexical decision experiment examining the transposed-letter effect in fluent braille readers. They compared error rates and latencies to pseudowords created by transposing two nonadjacent letters versus their corresponding substitution-letter controls (e.g., CHOLOCATE vs. CHOTONATE). They used the stimuli from an earlier lexical decision experiment that produced a sizeable transposed-letter effect in the visual modality (18.3% in the error data and 117.5 ms in the latency data; Carreiras et al., 2007). In contrast, they found no signs of a transposed-letter effect in braille. That is, transposed-letter pseudowords like CHOLOCATE are wordlike for sighted but not braille readers. Perea et al. (2012) interpreted this pattern as favoring those models that assume that the flexibility of letter position coding in the visual modality is due to perceptual uncertainty at locating objects in the space (i.e., as in the overlap model; Gomez et al., 2008) rather than a serial order mechanism shared by other modalities.

In a later experiment, Perea et al. (2015) examined the cost of reading sentences composed of intact braille words versus sentences in which two adjacent letters from some of the words were transposed. They also conducted a parallel experiment with sighted readers. Braille readers showed a substantially higher reading cost than sighted readers for the sentences with jumbled words. Nonetheless, braille readers could understand the phrases with jumbled words reasonably well, thus suggesting some flexibility in braille letter position coding, at least for the adjacent letter transpositions used in the experiment. However, one could raise two interpretive issues. First, participants were aware that several words in each sentence contained (adjacent) letter transpositions; hence they could have used context information to reconstruct the jumbled words. Second, the experiment did not include an orthographic replacement-letter control condition (i.e., the comparison was between intact vs. transposed conditions). The lack of this control condition makes it difficult to compare their finding with the large body of literature on transposed-letter effects.

One way to reconcile the above findings in braille with the ideas of the universality of serial order in cognitive tasks (see Fischer-Baum, 2018; Logan, 2021) is that braille readers show some noise regarding letter position coding during word recognition, but to a lesser extent than sighted readers. Indeed, one could argue that, due to the inherent seriality of braille, readers could show some flexibility in letter position coding for close, adjacent transpositions, but not for more distant, nonadjacent transpositions. Indirect evidence favoring this interpretation comes from a recent lexical decision experiment in another modality with an intrinsic serial nature: the auditory modality. Dufour and Grainger (2021) found phoneme transposition effects when the transpositions involved adjacent phonemes (e.g., SADRINE [baseword: sardine] produced slower responses than the control SAGLINE). Critically, they found no signs of a phoneme-transposition effect when the transpositions were nonadjacent (SARAFI [baseword: safari] produced similar response times and error rates as SALACHI). The findings from Dufour and Grainger (2021) suggest that the window of the flexibility of serial order is less for a modality in which the stimuli are perceived serially. Thus, the issue at stake in the present experiment is whether braille readers show a window of flexibility in letter position coding by examining the transposed-letter effect with adjacent letter transpositions.

Here, we designed a lexical decision experiment where the pseudowords were created by either transposing two adjacent letters (e.g., AVENIDA [avenue in Spanish] ➔ AVEINDA; LABORATORIO [laboratory] ➔ LABOARTORIO) or replacing two adjacent letters (orthographic controls; e.g., AVEARDA for AVEINDA; LABOESTORIO for LABOARTORIO). We used the same base words as in the Perea et al. (2012) experiment for comparison purposes. If the results show evidence in favor of a transposed-letter effect with adjacent letters (i.e., worse performance for LABOARTORIO than for LABOESTORIO), this will provide empirical support to the claim that the mechanisms behind serial order processes are universal (Fischer-Baum, 2018; Logan, 2021). This outcome would also favor those models of letter position where letter position encoding occurs at an amodal, orthographic level (e.g., Grainger & van Heuven, 2003). Contrarily, a similar performance for LABOARTORIO and LABOESTORIO in braille would pose problems to the said claim on the universality of serial order processing across modalities. Instead, this latter outcome would be more consistent with those models that assume that the flexibility in letter position coding in visual-word recognition originates from the uncertainty of locating visual objects (i.e., letters in visual-word recognition) to positions (e.g., Gomez et al., 2008).

## Method

This study was pre-registered on the Open Science Framework (OSF) before the start of data collection. The registration form, along with the materials, task scripts, data files, and analysis scripts, is available at https://osf.io/fdtv5/.

### Participants

The participants were 12 fluent braille readers, all of them native speakers of Spanish (seven female; mean age: 39.83 years; age range: 19–58). All participants were diagnosed with either severe visual impairment (four) or blindness (eight) at birth. In all cases, braille instruction started during their childhood (5–6 years old). Two of the participants were also taught to read with magnified printed letters. Regarding their educational level, two participants had completed high school, three were university students, five had a university degree, and two had a post-graduate degree. They were recruited with the help of the National Organization of Spanish Blind People (*Organización Nacional de Ciegos de España: ONCE).* All participants gave informed consent to participate and received a small monetary compensation (7.5€) for their participation in the study. The number of participants was determined via Sequential Bayes Factor Design (see Schönbrodt & Wagenmakers, 2018), as described in the pre-registration form for the study. After the first 12 participants, we computed the Bayes factor (BF) for the critical effects (i.e., transpositions vs. replacements for pseudoword data; high frequency vs. low frequency for word data) via a paired Bayesian *t*-test (with default priors) by subjects using the *BayesFactor* package (Morey & Rouder, 2014) in R (R Core Team, 2021). For word data, all BFs exceeded 3 (i.e., the established criterion in the pre-registration since it reflects evidence for/against an effect; see Lee & Wagenmakers, 2014) for accuracy and response time measures. For the pseudoword data, BF exceeded 3 for accuracy. It is important to note that response times in braille reading are long and highly variable (e.g., Lei et al., 2019; Perea et al., 2012; see also Bertelson et al., 1992), increasing the difficulty to disentangle the signal from the noise; hence, sampling stopped at n = 12.

### Apparatus

We used an *Active Braille* refreshable braille display (*Help Tech*; Saladino, 2019). This braille display was connected via USB to a Mac OS. We created a shell script to (1) present the stimuli on the braille display – having the OS-X's VoiceOver accessibility feature enabled, and (2) record the participant's responses. All the code is available in the Online Supplementary Material (see OSF repository).

### Materials

To create the pseudoword stimuli, we employed the 120 basewords from Perea et al. (2012) (mean length: 8.9 letters [range: 7–11]; mean frequency: 29.72 per million [range: 1.42– 212.30] in the Spanish EsPal database; Duchon et al., 2013). For each base word, we created a transposed-letter pseudoword by switching two internal, adjacent letters (e.g., transposition: AVENIDA [avenue] ➔ AVEINDA). For each transposed-letter pseudoword, we created a replacement-letter pseudoword by replacing the switched letters from the transposed-letter (e.g., the control for AVEINDA would be AVEARDA). The replacement letters were always consistent with the vowel/consonant status of the original letters. Bigram frequency was similar for transposed and replacement pseudowords (means = 1.96 vs. 1.94, respectively, derived from B-Pal; Davis & Perea, 2005).

For the word stimuli, we employed 120 Spanish words, 60 of which were high-frequency words (mean length: 8.92 letters [range: 7–11]; mean frequency: 3.09 per million [range: 0.38–6.85]) and the other 60 were low-frequency words (mean length: 8.92 letters [range: 7–11]; mean frequency: 106.14 per million [range: 30.40–823.45]).^2^

We created two counterbalanced lists (e.g., if AVEINDA were in List 1, AVEARDA would be in List 2; there were 60 items of each pseudoword condition in each list). The session started with 12 practice trials (six pseudowords + six words) to familiarize the participants with the task. All the experimental trials were presented in random order to each participant. All the stimuli are available in the OSF repository.

### Procedure

The experiment took place in a quiet room with one participant at a time. Participants were instructed to use the index finger of their preferred reading hand to perceive the stimuli presented in the braille display. They were asked to use their other hand to make a lexical decision (i.e., *Is the stimulus a Spanish word or not?*) as fast and accurately as possible by pressing the *word* or *non-word* key ("M" and "N", respectively) on the computer's keyboard. The stimuli remained in the braille display until a response was made. Response times were measured from each trial presentation onset. Inter-trial-interval (ITI) was 1.3 s – this allowed participants to reset their preferred finger to the beginning of the braille display. Each experimental session lasted around 30 min.

## Results

Trials in which responses were either longer than 8 s or shorter than 0.250 s – keep in mind that response times for braille word recognition are usually above 2 s (see Bertelson et al., 1992) – were excluded from the analysis (less than 1.6% of the data). This criterion was established before data collection. Table 1 summarizes the average participant performance in each condition.

**Table 1:** Mean accuracy (proportion) and response times (RTs) for correct and incorrect responses (in ms) for each condition

Lexicality	Type	Accuracy	RT correct	RT error
Pseudoword	Replacement	0.977	3117	3751
Pseudoword	Transposition	0.839	3231	2780
Word	High Frequency	0.978	2578	3484
Word	Low Frequency	0.947	2725	3261

**Fig. 1:**
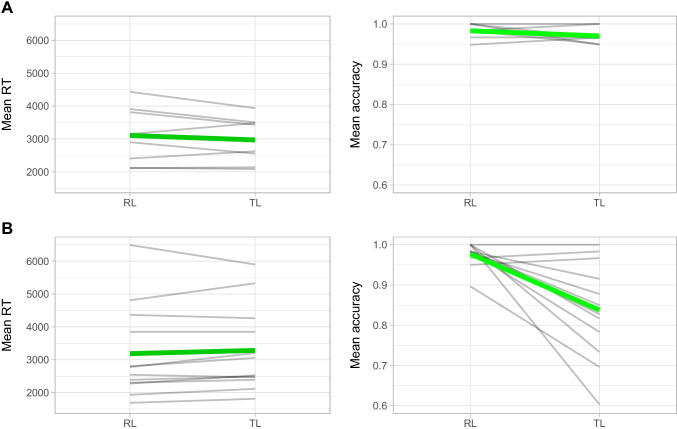
Mean response time (RT) and accuracy overall (in green) and by participant (in grey) for replacement-letter (RL) and transposed-letter (TL) pseudowords. The top two plots correspond to data from Perea et al. (2012). The bottom two plots correspond to the present experiment

**Fig. 2:**
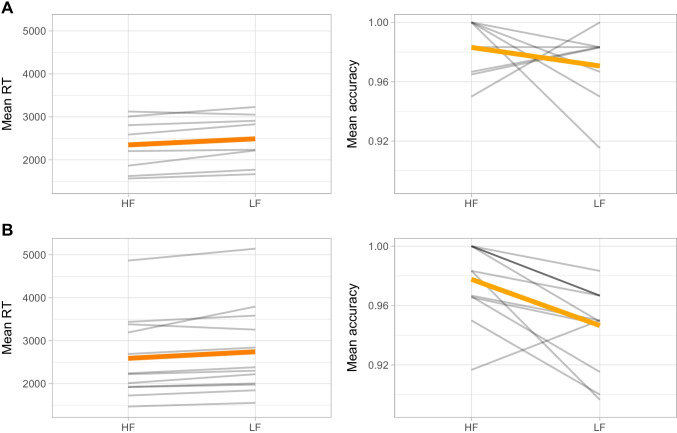
Mean response time and accuracy overall (in orange) and by participant (in grey) for high-frequency (HF) and Low-frequency (LF) words. The top two plots correspond to data from Perea et al. (2012). The bottom two plots correspond to the present experiment

To gain the full picture, it may be relevant to show the actual effects of the transposed letter versus those obtained by Perea et al. (2012). For that reason, we re-analyzed their data using the same analysis procedure that was pre-registered for the present experiment. Figures 1 and 2 show the by-participant transposed-letter effects in Perea et al.’s (2012) experiment with nonadjacent transpositions and the current experiment with adjacent transpositions.

### Pseudoword data

Correct response times and accuracy for pseudoword stimuli were analyzed separately using Bayesian linear mixed-effects models (brms package; Bürkner, 2017) in R (R Core Team, 2021). These models included *Type of pseudoword* (transposition vs. replacement; coded as -0.5 and 0.5, respectively) as fixed factor and *Subject* and *Item* as random factors (both intercepts and slopes). The ex-Gaussian link function was chosen for the latency analysis because it fits well the positive skew of the distribution of response times. The Bernoulli link function was chosen for the accuracy analysis, given the binary nature of this measure (correct (1) vs. incorrect (0)). All in all, the models for pseudoword data used the following syntax:$DependentVariable\;\sim\;Type\;of\;pseudoword\;+\;(1\;+\;Type\;of\;pseudoword\;\backslash\;Subject)\;+\;(1\;+\;Type\;of\;pseudoword\;\backslash\;Item)$ 

The models had four chains of 5,000 iterations each (1,000 as a warmup). Results from Bayesian linear mixed-effects models provide the value of each estimate, their standard error, and the 95% credible interval (95% CrI) of their posterior distributions. When the 95% CrI does not contain zero, it was taken as evidence in favor of an effect. All the analyses reported in this paper and additional analyses showing parallel results using frequentist linear mixed-effects models – which showed the same results – are available at the OSF repository.^3^

All the models converged, and all R̂ values were 1.00. The analysis of the accuracy data in the present experiment showed a transposed-letter effect: accuracy was higher for replacement pseudowords than for transposition pseudowords (97.7% vs. 83.9%, respectively), *b* = 2.60 *SE* = 0.84, 95% CrI [1.12, 4.47]. The parallel analysis on Perea et al. (2012) data did not show evidence of an effect (*b* = 0.68, *SE* = 0.69, CrI [-0.61, 2.13]) – note that the data followed the same trend (see the left plot in Panel A of Fig. 1).

The analysis of the correct response time data showed an effect in the same direction as the analysis of the accuracy data: responses were faster for replacement than for transposition pseudowords (3,117 ms vs. 3,231 ms, respectively); although the evidence was not as straightforward to establish an effect, *b* = -86.94, *SE* = 77.21, CrI [-242.80, 67.24].^4^ Perea et al. (2012) data did not show a transposed-letter effect either (*b* = 75.96, *SE* = 109.58, CrI [-134.84, 306.63]; note that, if anything, the effect was in the opposite direction (see the right plot in Panel A of Fig. 1).

### Word data

The analyses were parallel to those of the pseudoword data, except that the models included *Word frequency* (low vs. high; coded as -0.5 and 0.5, respectively) as a fixed factor and *Subject* and *Item* as random factors (both intercepts and slopes for subjects, only intercept for item). The models for pseudoword data used the following structure:

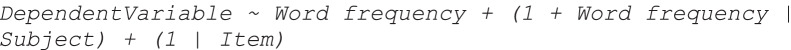



The models had four chains of 5,000 iterations each (1,000 as a warmup), and again, the ex-Gaussian link function was chosen for the latency analysis, and the Bernoulli link function was chosen for the accuracy analysis. All models converged ($\hat{R}$  = 1.00 for all models).

As depicted in Fig. 2, our results replicated Perea et al. (2012), showing a word-frequency effect. High-frequency words were classified more accurately (*b* = 1.08, *SE* = 0.44, 95% CrI [0.31, 2.06]) and faster (*b* = -95.87, *SE* = 40.33, 95% CrI [-176.27, -18.73]) than low-frequency words. The data from Perea et al. (2012) showed an effect in response times (*b* = -100.64, *SE* = 39.19, CrI [-178.39, -24.20]) – the effect on accuracy was in the same direction but its estimate crossed zero (*b* = 0.70, *SE* = 0.94, CrI [-1.12, 2.64]).^5^

## Discussion

Whether or not there are common cognitive mechanisms across modalities and cognitive domains to encode serial order is paramount in cognitive psychology (see Fischer-Baum, 2018; Logan, 2021). A benchmark phenomenon in the visual-word recognition literature is that transposed-letter pseudowords (e.g., JUGDE, CHOLOCATE) generate a percept much more similar to their base words than replacement-letter controls (e.g., JUPTE, CHOTONATE). This transposed-letter effect, which has been taken as a marker of the flexibility of letter position coding, is a fundamental element of the front-end of all current models of visual-word recognition (e.g., Adelman, 2011; Davis, 2010; Gomez et al., 2008; Grainger et al., 2006; Grainger & van Heuven, 2003; Norris & Kinoshita, 2012) and sighted reading (see Reichle, 2021). Notably, a prior experiment in the tactile modality did not find any signs of the transposed-letter effect for nonadjacent transpositions with braille readers (Perea et al., 2012). This pattern was attributed to an alleged qualitatively different processing of letter position coding in braille, thus limiting the idea of a common mechanism for coding serial order. Here, we tested the hypothesis that the core mechanisms behind the encoding of serial order in orthographic processing are fundamentally similar across modalities by transposing adjacent letter positions in a braille lexical decision task.

Our results showed that the responses to transposed-letter pseudowords were much less accurate than their corresponding replacement-letter controls (the effect was 13.8%). The response time data showed the same trend (the difference was 114 ms). This sizeable transposed-letter effect with adjacent transpositions favors the idea of a shared processing mechanism to encode letter position coding in words and probably serial order in general (see Fischer-Baum, 2018; Houghton, 2018; Logan, 2021). Moreover, this pattern is consistent with those neuroimaging studies showing that reading in both braille and print activates the same anatomical areas (i.e., the “Visual Word Form Area”; e.g., Reich et al., 2011). At the same time, the lack of an effect for nonadjacent letter transpositions in braille (Perea et al., 2012; re-analyzed in the present paper) and nonadjacent phoneme transpositions in the auditory modality (Dufour & Grainger, 2021) suggests that the characteristics of the sensory modality that receives the information modulate serial order processing. These findings add to the view that the flexibility of serial order during word processing, while universal, is not fixed. Instead, different variables can shape its extent, including the characteristics of each language (e.g., see Frost, 2012, and Perea et al., 2018, for evidence in Hebrew and Thai, respectively) and the participants’ reading abilities (see Gomez et al. 2021).

Thus, our findings are consistent with orthographic accounts of letter position coding proposed (e.g., Grainger & van Heuven, 2003). The only quantitative change is the extent of the flexibility of the open bigrams. Braille readers would only activate contiguous and contiguous + 1 open bigrams (i.e., AVENIDA, AV-AE-VE-VN-EN-EI-NI-ND-ID-IA-DA). As a result, adjacent transposed-letter pseudowords (e.g., AVEINDA: AV-AE-VE-VI-EI-EN-IN-ID-ND-NA-DA, eight shared bigrams with its baseword) would be more similar to their base word than replaced-letter pseudowords (AVEORDA: AV-AE-VE-VO-EO-ER-OR-OD-RD-RA-DA; four shared bigrams). Critically, the parallel difference is minimal when the transpositions are nonadjacent (ANEVIDA: AN-AE-NE-NV-EV-EI-VI-VD-ID-IA-DA, five shared bigrams; ARESIDA; AR-AE-RE-RS-ES-EI-SI-SD-ID-IA-DA, four shared bigrams).

Perceptual models of word recognition can also accommodate the findings in braille with some minor modifications. While there is some flexibility in letter position coding in braille, the perceptual noise associated with each letter position would be much narrower than in the visual modality. We acknowledge, however, that this explanation cast some doubts on whether the limitations of the visual system are the primary cause behind letter transposition effects, as initially proposed by perceptual models of letter position coding (e.g., Gomez et al., 2008).

In sum, the present study offers a critical piece to the letter position coding puzzle, showing sizeable transposed-letter effects with adjacent transpositions in the tactile modality with braille readers. These findings suggest that the inherent serial nature of the tactile modality – concerning language processing – reduces, but does not eliminate, the flexibility of serial order during word recognition. Therefore, these findings favor a common, domain-general mechanism of serial order shaped by the constraints posed by the specific sensory modality at play.

## Footnotes

1 There are many ways of reading in braille. Readers can use just one hand or two hands; and, within each hand, they can use just one finger or multiple fingers. Moreover, there are different movement techniques for those using two hands (regardless of the number of fingers). For instance, *left marks*, in which the right hand reads and the left hand keeps its place at the beginning of the line; or *scissors, in which* the left hand reads to the middle of the line and the right hand continues after that till the end of the line while the left hand waits at the beginning of the next line (e.g., Wright et al., 2009). For the purposes of the present paper, we only describe one-finger reading. Nonetheless, as a reviewer noted, braille reading yields a more serial sensory input than print reading for all reading strategies.

2 Most of these words (70%) were taken from Perea et al. (2012) experiment. We did not employ all the items in their experiment because the frequencies of some of their words in the updated database we employed (EsPal; based on subtitles) were outside the low- or high-frequency range. This database has been a better predictor of behavioral responses than the older lexical databases (Duchon et al., 2013).

3 In the Online Supplementary Material, we included analyses showing that the differences in the pattern of transposed-letter effects between the Perea et al. (2012) experiment and the present experiment are not due to the characteristics of the participants.

4 Note that the mass of the distribution below 0 was less than 20%. The results of parallel analysis using the lmer function (lme4 package, Bates et al., 2015; and lmerTest package, Kuznetsova et al., 2017) with a standard -1000/RT transformation showed a significant effect (*b =* -0.0169*, SE=* 0.007, *t =* -2.322, *p =* 0.0303). This was not the case for the data from Perea et al. (2012), *b* = 0.005, *t* = 0.57, *p* = 0.586).

5 The comparison of the analysis of the word data must be done with some caution because the set of words was not exactly the same. As the current experiment employs a more updated Spanish database (Duchon et al., 2013), we replaced a small subset of words (30%) that did not comply with the criterion of “high-” and “low-” frequency words.
